# Membranous obliterans bronchitis after hematopoietic stem cell transplantation: case report

**DOI:** 10.1186/s12890-026-04539-x

**Published:** 2026-08-01

**Authors:** Weiquan Pan, Yang Li, Changjin Wei, Zhiyi He, Jianming Luo, Guopeng Liao, Lingyun Chen, Meihua Li

**Affiliations:** 1 Guangxi Medical University, Nanning, Guangxi Zhuangzu Autonomous Region 530021 P. R. China; 2 The First Affiliated Hospital of Guangxi Medical University, Nanning, Guangxi Zhuang Autonomous Region 530021 P.R. China

**Keywords:** Hematopoietic stem cell transplantation, Bronchiolitis obliterans, Graft-versus-host disease, Fiberoptic bronchoscopy

## Abstract

**Background:**

Pulmonary complications following hematopoietic stem cell transplantation (HSCT) are crucial factors affecting the prognosis of pediatric patients. Among them, membranous obliterative bronchitis (MOB) is rare, lacks sufficient clinical recognition, and is easily confused with bronchiolitis obliterans (BO), often leading to delayed diagnosis and extremely poor treatment outcomes. This study aimed to clarify the clinical characteristics, diagnostic key points of MOB after HSCT and its association with graft-versus-host disease (GVHD), so as to bridge the current knowledge gap regarding this rare fatal complication in clinical practice.

**Methods:**

A retrospective analysis was conducted, enrolling 2 pediatric patients with severe β-thalassemia who were diagnosed with MOB after HSCT. Data including clinical manifestations, chest computed tomography (CT), pulmonary function, fiberoptic bronchoscopy, pathological biopsy, metagenomic next-generation sequencing (mNGS) of bronchoalveolar lavage fluid, and immune indicators were systematically collected. The diagnosis and treatment processes were summarized, and the pathogenesis was analyzed. The 2 patients developed progressive dyspnea at 7 months and 4 months after HSCT, respectively, both presenting with severe obstructive ventilatory dysfunction in pulmonary function tests (FEV1% predicted: 23.2% and 22.1%, respectively). Fiberoptic bronchoscopy revealed characteristic membranous stenosis or occlusion in segmental/subsegmental bronchi (3rd–4th generation), and bronchiectasis was observed on chest CT in both cases. Both patients were complicated with active GVHD and opportunistic infections. The conditions could not be reversed despite immunosuppressive therapy, anti-infective treatment, and interventional fiberoptic bronchoscopy, and both eventually died of respiratory failure. Immune monitoring indicated excessive activation of CD8 + T cells, persistently lowered CD4/CD8 ratio, and decreased B cell counts.

**Conclusions:**

MOB after HSCT is a rare fatal complication that may be closely related to GVHD progression, immune dysregulation, and infectious triggers. Membranous occlusion under fiberoptic bronchoscopy is the key to definitive diagnosis, and existing therapeutic measures have limited efficacy. Clinically, early fiberoptic bronchoscopy should be performed for dyspneic patients complicated with GVHD after HSCT, and the prevention and control of GVHD as well as infection prophylaxis should be strengthened. Targeted immune intervention strategies should be explored in the future to improve prognosis.

## Introduction

Hematopoietic stem cell transplantation (HSCT) is a core treatment for curing malignant hematological diseases and some hereditary hematological disorders. Having been performed in China for more than 30 years, the technique has been continuously updated with steadily improved therapeutic effects, and has become an important part of the diagnosis and treatment system for hematological diseases [1]. However, various complications after transplantation remain a key factor restricting patients’ long-term survival and quality of life. Among them, pulmonary complications have a high incidence and strong lethality, representing a major difficulty in clinical management [2].

Under long-term immunosuppression, infectious pulmonary complications were once the main pulmonary risks after HSCT. With advances in etiological detection technologies and the standardized application of broad-spectrum antimicrobial agents, such complications have been effectively controlled. non-infectious pulmonary complications remain prominent, with poor understanding of their pathogenesis and treatment paradigms. Clinicians ought to strengthen surveillance and implement early interventions without delay [3].

Bronchiolitis obliterans (BO) is the most common delayed non-infectious pulmonary complication after HSCT, falling under the category of chronic pulmonary graft-versus-host disease (cGVHD). It is characterized by obstructive lesions of the small airways, with relatively in-depth clinical diagnosis, treatment and research, and well-established understanding [4]. In contrast, obliterans bronchitis (OB) is a rare large-airway obliterative disease that mainly affects medium and small cartilaginous bronchi, divided into classical and membranous subtypes (membranous obliterans bronchitis, MOB). Among them, MOB is clinically rare, characterized by membranous polypoid occlusion of the lumen of segmental and subsegmental bronchi, and is easily confused with BO [5].

At present, there are very few studies on MOB after HSCT at home and abroad, most of which are scattered case reports or small-sample case series. Clinicians lack sufficient understanding of its clinical features, diagnostic criteria and pathogenesis, leading to frequent misdiagnosis, missed diagnosis and delayed intervention. Based on this, this study reports 2 confirmed cases of MOB after HSCT. Combined with the patients’ immune monitoring, imaging and pathological data, it summarizes the clinical characteristics and diagnostic basis of MOB after HSCT, explores the potential role of cGVHD-related immune disorders in its pathogenesis, and calls for the early implementation of fiberoptic bronchoscopy in the evaluation of dyspneic patients after HSCT, so as to provide clinical references for the early identification and precise intervention of such highly lethal complications.

## Case presentation

### Case 1

General Data: Male, 12 years old, admitted to hospital on January 1, 2021, due to “cough, wheezing and diarrhea for 3 days”. Primary Disease and Transplant History: Diagnosed with severe β-thalassemia, genotype 41-42 M/-28 M; laparoscopic splenectomy performed on September 12, 2019; bone marrow plus peripheral blood stem cell transplantation performed on May 8, 2020 (father-to-son donation, ABO blood group identical, 7/12 HLA high-resolution matching); donor lymphocyte infusion performed on October 30, 2020; acute cutaneous GVHD diagnosed on November 10, 2020, which remitted after anti-rejection therapy with methylprednisolone.

Present Illness and Physical Examination: On December 29, 2020, the patient developed cough and wheezing without obvious inducement, accompanied by expectoration and diarrhea (more than 10 episodes of yellow-green watery stool per day), without fever, chest pain or hemoptysis. Physical examination on admission: body temperature 36.4℃, pulse 135 beats/min, respiratory rate 32 breaths/min, blood pressure 121/95 mmHg; acute facies, clear consciousness, positive inspiratory three-depressions sign; coarse bilateral breath sounds with numerous wheezes and fine crackles; abdominal distension, liver palpable 6 cm below the costal margin with soft consistency; digital clubbing of extremities, no other abnormal findings.

**Fig. 1:**
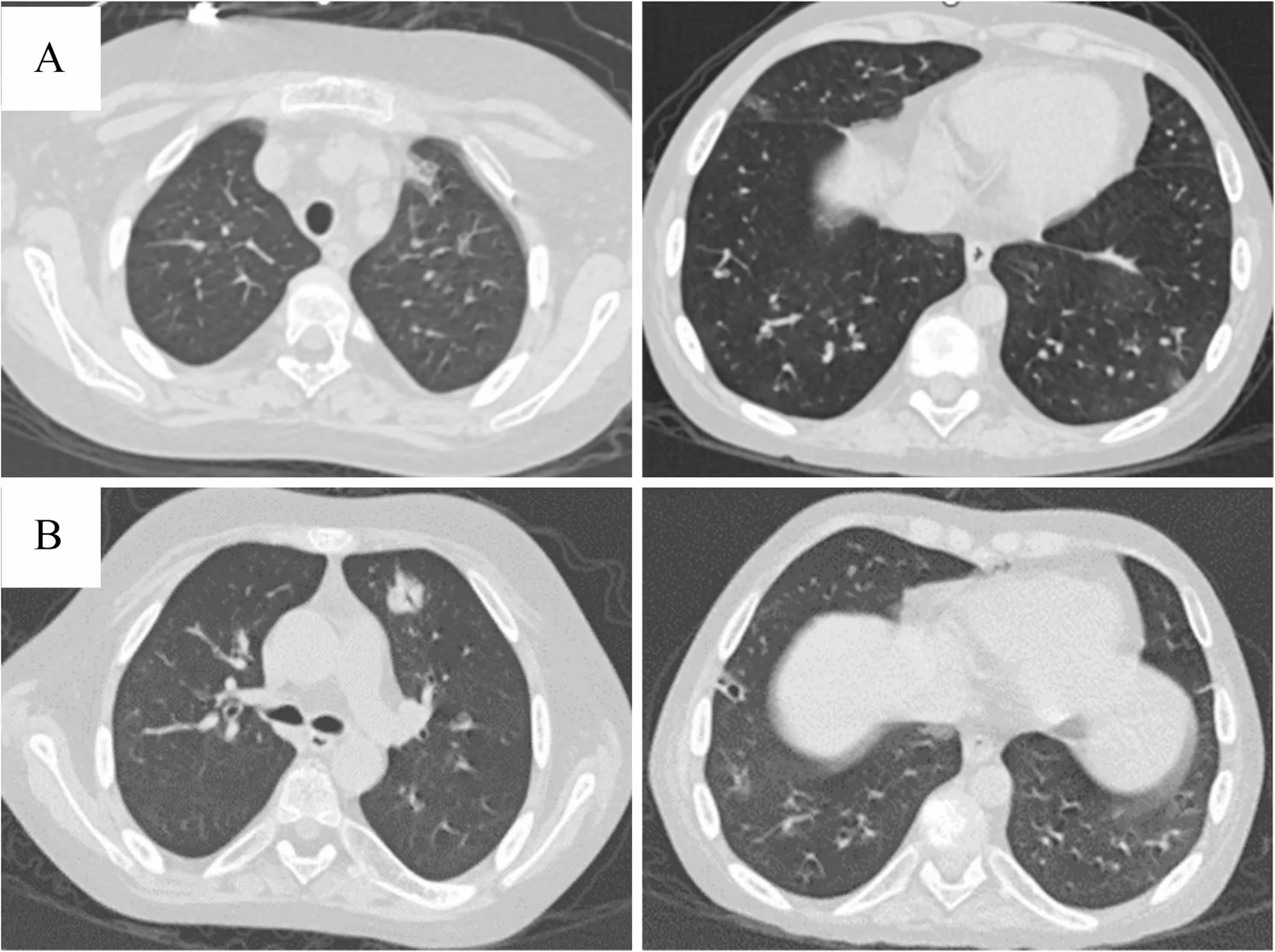
Chest CT of case 1. **A**: (January 1, 2021) Multiple flocculent increased-density shadows are seen in both lungs with blurred borders, suggesting inflammation in both lungs. A solid nodule approximately 0.6 cm in diameter with clear borders is observed in the dorsal segment of the right lower lobe, considered an inflammatory nodule. **B**: (January 23, 2021) Multiple patchy and streaky increased-density shadows are seen in both lungs, which are increased compared with the previous examination. Air bronchograms are visible in the anterior segment of the left upper lobe. Cylindrical and cystic bronchiectasis is found in the medial segment of the right middle lobe, as well as the anterior basal and lateral basal segments of the right lower lobe

**Fig. 2:**
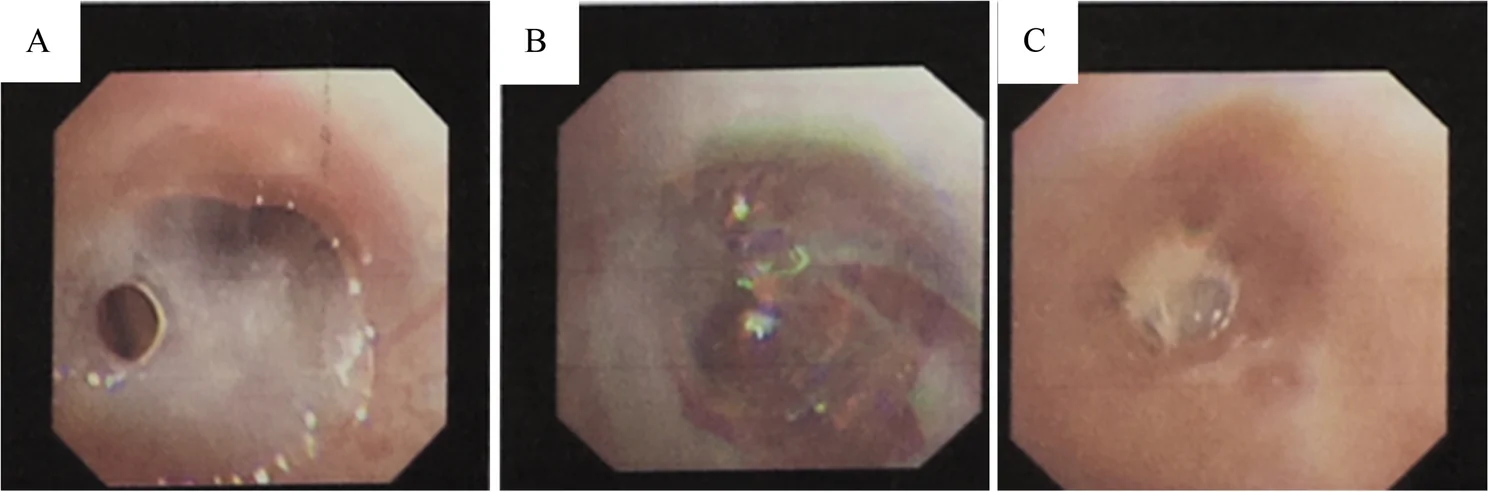
Bronchoscope of case 1. **A**: Membranous stenosis of the right lower lobe segmental bronchus; **B**: Membranous occlusion of the subsegmental bronchus; **C**: Membranous scar occlusion of the right lower lobe segmental bronchus

**Fig. 3:**
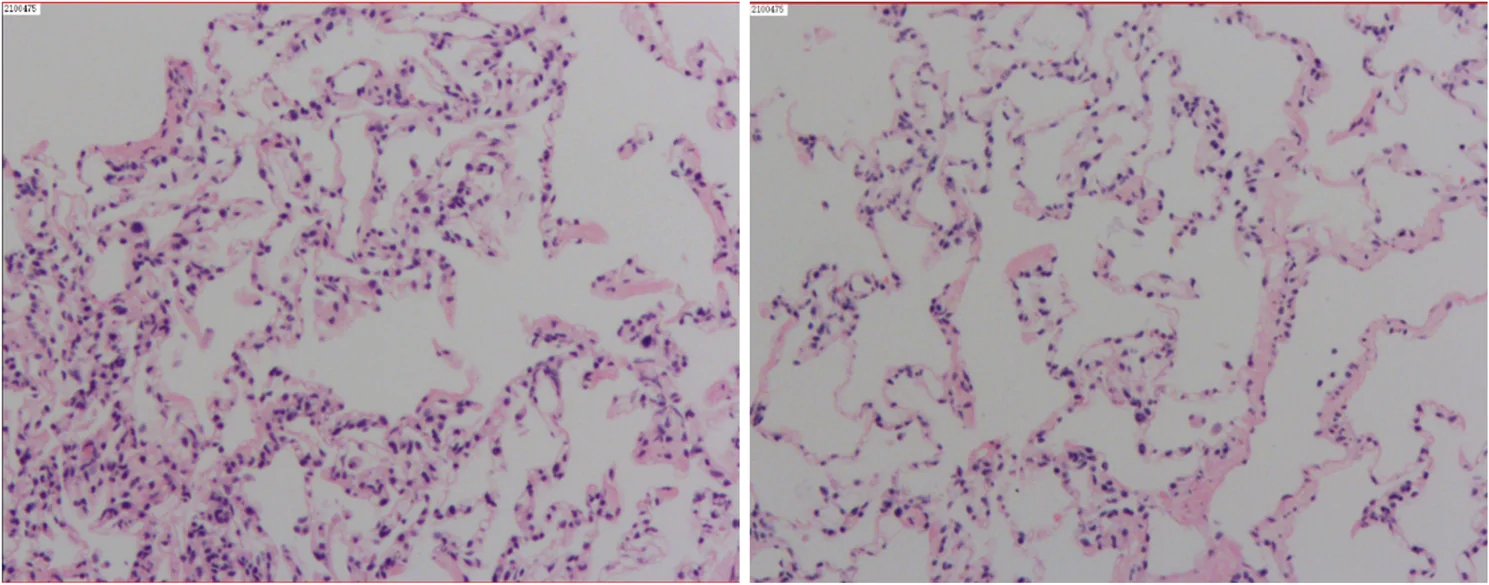
Histopathology of case 1. (TBLB) Pathological examination: (bronchial) mucosal chronic inflammation. No granulation tissue filling was observed in the alveolar spaces. No bronchioles were identified

**Fig. 4:**
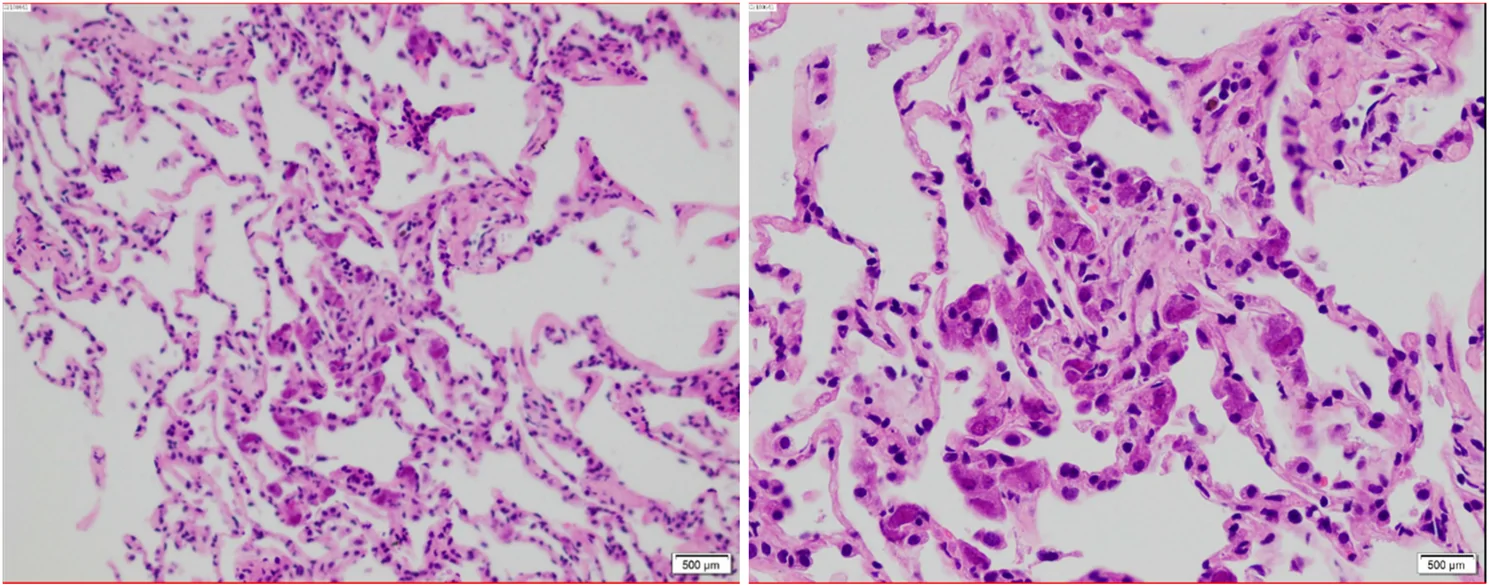
Histopathology of case 1. (Percutaneous Pulmonary Biopsy) Pathological Examination: The alveolar spaces are patent, the alveolar septa are slightly widened, and viral inclusions are visible. No vascular endovasculitis or necrosis is observed. The alveolar spaces are not filled with granulation tissue. No bronchioles are identified

Auxiliary Examinations: ① Blood gas analysis (2021-01-01): pH 7.491, PaO₂ 70 mmHg, PaCO₂ 41.7 mmHg, oxygenation index 280; ② Routine blood test: white blood cells 7.05 × 10⁹/L, hemoglobin 106.3 g/L, platelets 111.50 × 10⁹/L, absolute neutrophil count 3.41 × 10⁹/L; ③ Inflammatory markers: procalcitonin 0.202 ng/ml, C-reactive protein 39.66 mg/L; ④ Immune function: IgG 4.54 g/L, IgA 0.4 g/L, IgM 0.24 g/L; 2020-08-10: Total T cells 88.39%, CD4 + T cells 15/µl (2.39%), CD8 + T cells 498/µl (80.5%), CD4/CD8 ratio 0.03, B cells 0.26%, NK cells 8.75%; 2020-11-02: Total T cells 62.18%, CD4 + T cells 328/µl (29.96%), CD8 + T cells 314/µl (28.71%), CD4/CD8 ratio 1.04, B cells 11.55%, NK cells 24.36%; 2021-01-28: Total T cells 46.6%, CD4 + T cells 44/µl (13.83%), CD8 + T cells 114/µl (35.44%), CD4/CD8 ratio 0.39, B cells 0.99%, NK cells 55.07%; ⑤ Etiology: cytomegalovirus DNA 1.15 × 10³ copies/ml, sputum culture showed normal pharyngeal flora, fungal G test, aspergillus GM test, respiratory pathogen IgM, and Epstein-Barr virus nucleic acid were all negative; ⑥ Chest CT (2021-01-01): multiple flocculent inflammatory infiltrates in both lungs, 0.6 cm-diameter inflammatory nodule in the dorsal segment of the right lower lobe (Fig. 1-A); ⑦ Pulmonary function (2021-01-11): severe obstructive combined with moderate restrictive ventilatory dysfunction (FEV1%pred 23.2%, FVC%pred 53.9%, FEV1/FVC 36.06%, severe impairment of small airway ventilatory function), negative bronchial dilation test; ⑧ Fiberoptic bronchoscopy (2021-01-14): thick secretions observed in all lobar and segmental bronchi of both lungs; after lavage, membranous stenosis/occlusion of multiple segmental and subsegmental bronchi (grades 3–4) (consistent with MOB features, Fig. 2); ⑨ Pathological examination: transbronchial cryobiopsy (TBLC) showed chronic inflammation of bronchial mucosa; percutaneous lung biopsy showed widened alveolar septa with viral inclusions, no vascular endovasculitis or necrosis (Figs. 3 and 4); ⑩ Bronchoalveolar lavage fluid (BALF): cytological classification showed neutrophils 5%, lymphocytes 25%, histiocytes 70%; mNGS detected Aspergillus fumigatus, cytomegalovirus, etc., with negative bacterial and fungal cultures.

Admission Diagnosis: Obliterative bronchitis (OB), pulmonary infection, cytomegalovirus viremia, acute gastrointestinal GVHD.

Treatment Course and Outcome: Empirical anti-infective therapy, anti-GVHD therapy with methylprednisolone (3–4 mg·kg⁻¹·d⁻¹) combined with mycophenolate mofetil and tacrolimus, non-invasive ventilation, etc., were administered; interventional therapy under fiberoptic bronchoscopy (biopsy forceps dilation plus occlusive membrane fenestration) performed on January 20, 2021; follow-up chest CT on January 23, 2021 showed aggravated pulmonary inflammation and new-onset bronchiectasis (Fig. 1-B). The condition progressed continuously with secondary multiple organ dysfunction, and the patient died in the pediatric intensive care unit on February 1, 2021 (31 days after admission).

### Case 2

General Data: Male, 11 years old, admitted to hospital on April 1, 2021, due to “cough and dyspnea for 2 days”. Primary Disease and Transplant History: Diagnosed with severe β-thalassemia, genotype 71-72 M/IVS-I-1 M; peripheral blood stem cell transplantation performed on November 18, 2020 (unrelated donor, ABO blood group identical, full HLA matching); cutaneous and gastrointestinal aGVHD occurred in December 2020, which remitted after intensive anti-rejection therapy with methylprednisolone, mycophenolate mofetil, ruxolitinib, sirolimus, basiliximab, etc., while diarrhea persisted for more than 2 months.

Present History and Physical Examination: On March 30, 2021, the patient developed cough and dyspnea on exertion without inducement, accompanied by fatigue, without fever, chest pain or hemoptysis. Physical examination on admission: body temperature 36.3℃, pulse 86 beats/min, respiratory rate 20 breaths/min, blood pressure 97/74 mmHg; clear consciousness, chronic facies, positive inspiratory three-depressions sign; coarse bilateral breath sounds, no dry or moist crackles or pleural friction rub; digital clubbing of extremities. No other obvious abnormal findings.

**Fig. 5:**
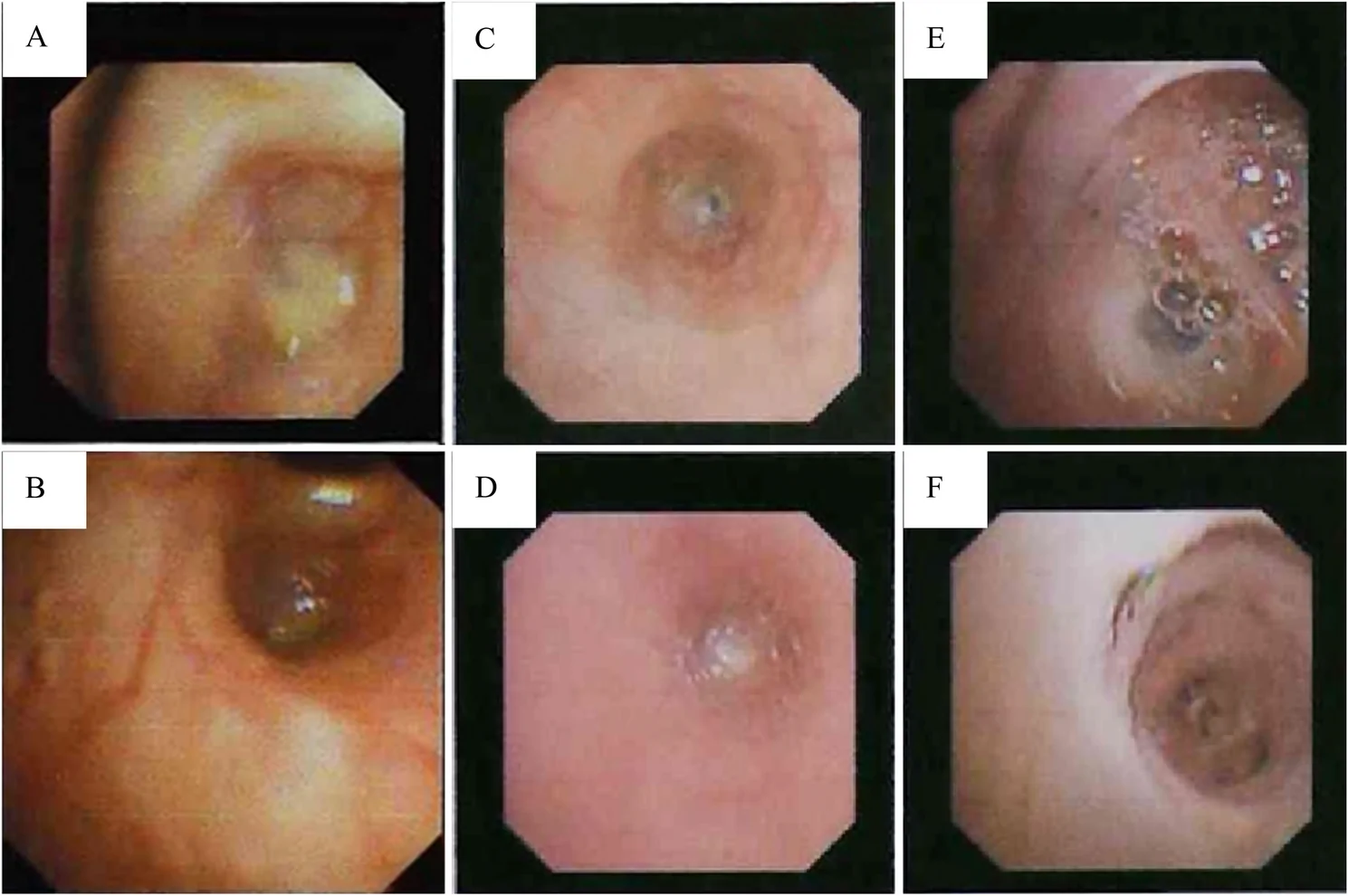
Bronchoscope of case 2. 2021-04-02:**A**, **B**: Membranous occlusion in multiple segmental and subsegmental bronchi (3rd to 4th order) of both lungs. 2021-07-14:**C**, **D**: Membranous occlusion of a subsegmental bronchus in the right lower lobe; **E**: Membranous stenosis or occlusion of a subsegmental bronchus in the left upper lobe; **F**: Newly identified membranous occlusion of a subsegmental bronchus in the right upper lobe

**Fig. 6:**
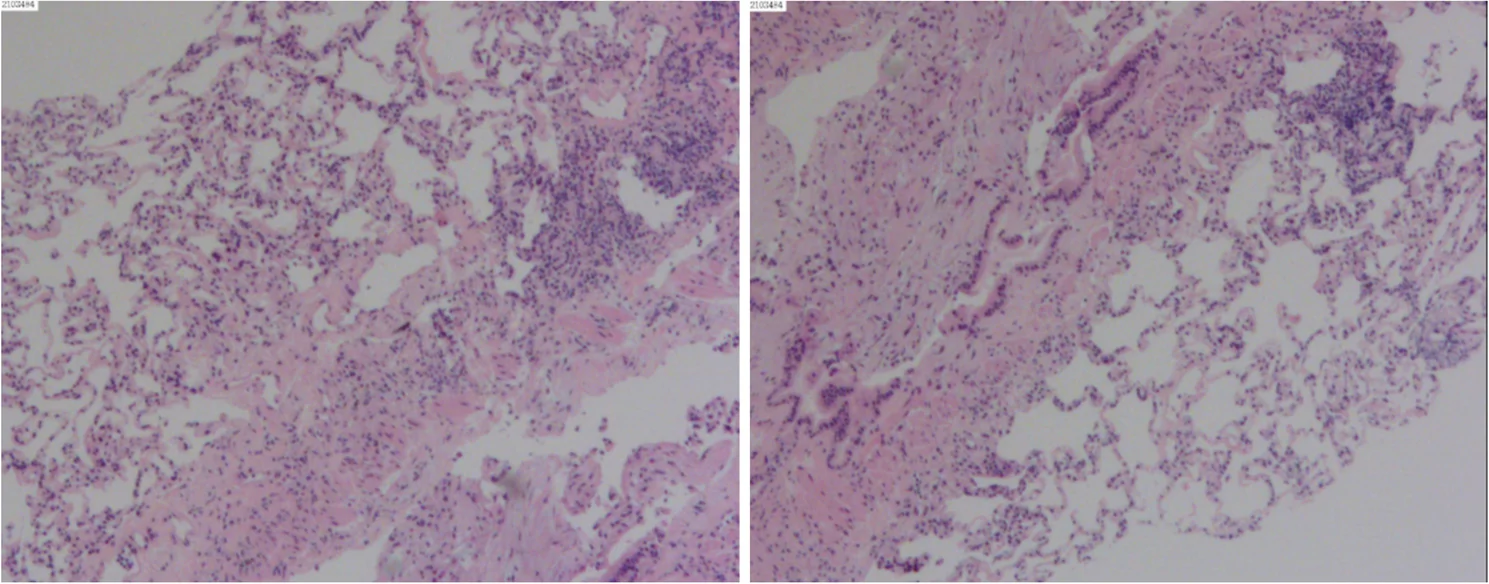
Histopathology of case 2. (Pathological examination of transbronchial cryobiopsy): (Frozen sections of lung tissue) The submitted tiny bronchial mucosa and lung tissue show focal infiltration of numerous lymphocytes, indicating chronic inflammatory changes. No obliteration is observed in the bronchioles, and no filling of granulation tissue is seen in the alveolar spaces

Auxiliary Examinations: ① Routine blood test: white blood cells 2.08 × 10⁹/L, hemoglobin 109.7 g/L, platelets 98.4 × 10⁹/L, absolute neutrophil count 1.07 × 10⁹/L; ② Inflammatory markers: C-reactive protein 16 mg/L, interleukin-6 9.1 pg/ml, procalcitonin 0.041 ng/ml; ③ Immune function: 2020-11-02: Total T cells 76.83%, CD4 + T cells 845/µl (36.18%), CD8 + T cells 825/µl (35.34%), CD4/CD8 ratio 1.02, B cells 17.6%, NK cells 4.23%; 2020-12-15: Total T cells 77.67%, CD4 + T cells 65/µl (7.78%), CD8 + T cells 550/µl (66.02%), CD4/CD8 ratio 0.12, B cells 2.04%, NK cells 21.56%; 2021-07-19: Total T cells 84.57%, CD4 + T cells 30/µl (8.33%), CD8 + T cells 255/µl (69.71%), CD4/CD8 ratio 0.18, B cells 3.2%, NK cells 11.72%; ④ Etiology: sputum culture showed pharyngeal flora, cytomegalovirus DNA, Epstein-Barr virus nucleic acid, and Mycoplasma pneumoniae antibody were all normal; ⑤ Chest CT (2021-03-31): solid micronodule in the lateral segment of the right middle lobe; ⑥ Pulmonary function (2021-04-01): severe obstructive combined with severe restrictive ventilatory dysfunction (FEV1%pred: 22.1%, FVC%pred: 40.3%, FEV1/FVC: 46.16%, MEF25: 9.4%, MEF50: 7.2%, MEF75/25: 7%), negative bronchial dilation test; ⑦ Fiberoptic bronchoscopy (2021-04-02): membranous occlusion of multiple segmental and subsegmental bronchi (grades 3–4) in the right middle and lower lobes, left upper and lower lobes (Fig. 5); ⑧ Pathological and lavage fluid examinations: TBLC (right lower lobe) showed local massive lymphocytic infiltration in the tiny bronchial mucosa and lung tissue, showing chronic inflammatory changes (Fig. 6); BALF cytological classification: neutrophils 50%, lymphocytes 30%, histiocytes 19%, eosinophils 1%; BALF culture showed pharyngeal flora.

Admission Diagnosis: Obliterative bronchitis (OB), pulmonary infection.

**Fig. 7:**
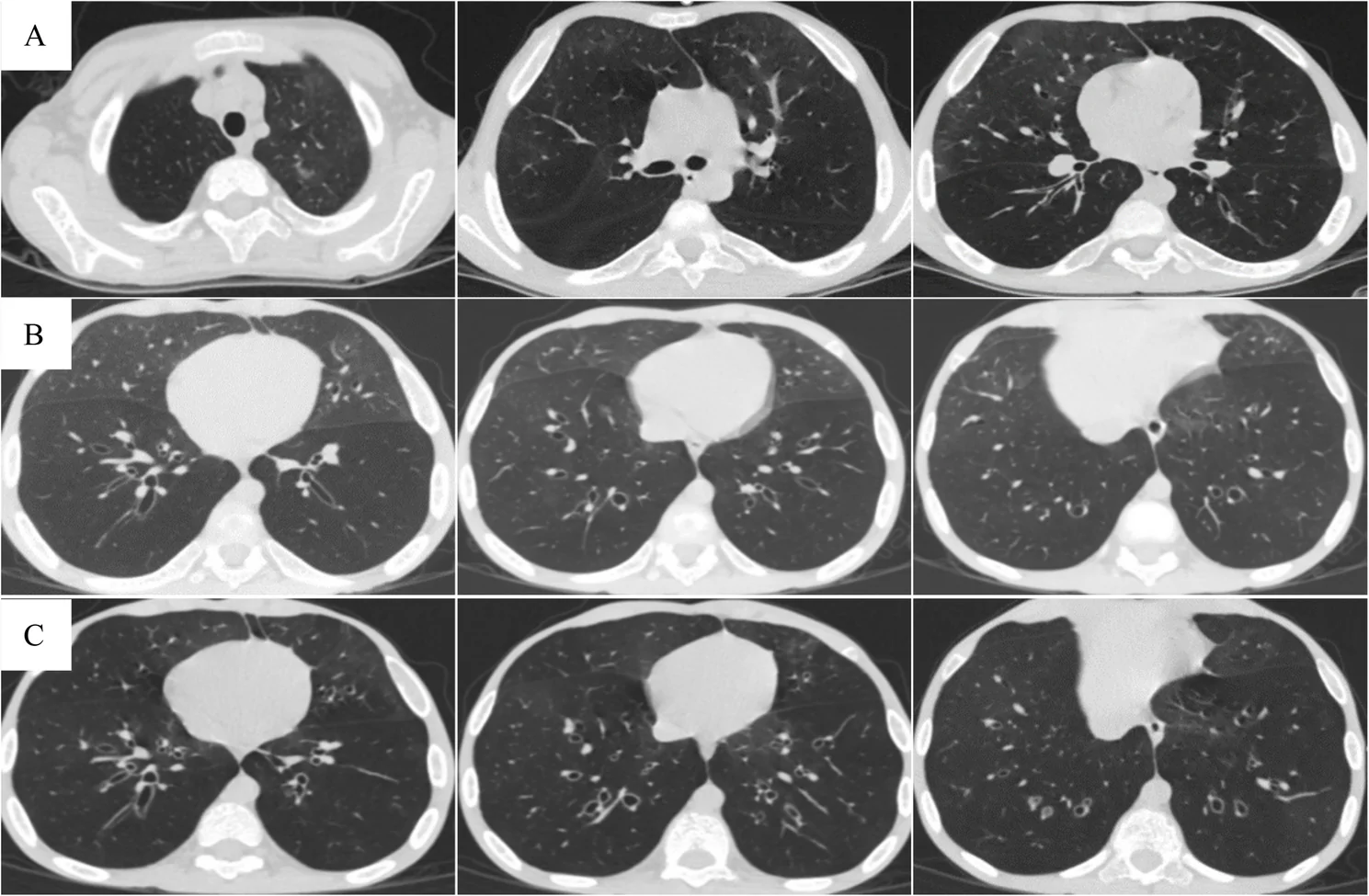
Chest CT of case 2. **A**: (2021-06-24) Patchy and flocculent high-density shadows are seen in all lobes of both lungs with blurred margins, suggesting pneumonia. **B**: (2021-08-29) Patchy high-density shadows are observed in all lobes of both lungs with blurred margins; new partial bronchiectasis is noted. **C**: (2021-10-08) The lesions in both lungs have regressed compared with previous examinations, but some bronchi are more dilated than before

Treatment Course and Outcome: Initial treatment included empirical anti-infective therapy, anti-rejection therapy with methylprednisolone (2 mg/kg/d) combined with mycophenolate mofetil and sirolimus, supplemented by budesonide inhalation, azithromycin, montelukast and symptomatic supportive care, with transient improvement of symptoms; follow-up pulmonary function on May 6, 2021 showed further decrease in FEV1; cough and wheezing aggravated with yellow-white mucoid expectoration in late June 2021, without fever; follow-up chest CT on June 24, 2021 showed multiple patchy and flocculent fuzzy inflammatory opacities in both lungs (Fig. 7-A); follow-up fiberoptic bronchoscopy on July 14, 2021 showed new membranous occlusion of subsegmental bronchi in the right upper lobe, with other lesions unchanged (Fig. 5); Pneumocystis jirovecii was detected by BALF-mNGS (identification confidence 99%, sequence count 610), and targeted anti-Pneumocystis therapy was added immediately; follow-up chest CT on August 29, 2021 showed persistent inflammatory lesions in both lungs with new partial bilateral bronchiectasis (Fig. 7-B); follow-up fiberoptic bronchoscopy on September 1, 2021 showed no significant change in the extent of small bronchial occlusion; follow-up chest CT on October 8, 2021 showed absorption of pulmonary lesions compared with previous, but aggravated bronchiectasis (Fig. 7-C). The condition deteriorated sharply 8 months after onset, and the patient died in late November 2021.

## Discussion and conclusions

### Pathogenesis of MOB after HSCT

#### Graft-versus-host disease as a likely core inducing factor

Current evidence indicates MOB may be a complication of GVHD, and it may represent a specific manifestation of severe pulmonary GVHD. Yokoi et al. noted that bronchial mucosal glands serve as an important barrier for airway defense; mild glandular GVHD only involves the distal bronchioles, whereas severe glandular GVHD can extend lesions to cartilaginous segmental and subsegmental bronchi [6]. Both pediatric patients in this study developed multi-organ aGVHD involving the skin and gastrointestinal tract after transplantation. Based on our observations, MOB occurred in both cases after poor control of GVHD, which further supports the hypothesis that MOB may stem from progressive worsening of pulmonary aGVHD, rather than being an entirely independent complication. This finding suggests MOB may share similar allogeneic immune-related pathogenesis with extrapulmonary GVHD, although definitive confirmation requires further evidence.

#### Immune imbalance participates in the pathological process

Imbalance of T-cell subsets may serve as the immune mechanism underlying MOB. Both pediatric patients in this study exhibited characteristic immune disorders highly synchronized with disease progression. These immune abnormalities suggest that excessive activation of CD8 + cytotoxic T cells directly damages bronchial epithelial cells by releasing perforin and granzyme. Severe depletion of CD4 + T cells leads to defective regulatory T-cell function and disrupted immune homeostasis, which in turn promotes massive release of pro-inflammatory cytokines such as IFN-γ and TNF-α, as well as pro-fibrotic factors including TGF-β. Among them, TGF-β can activate fibroblasts and induce epithelial-mesenchymal transition, directly driving the formation of fibrous membranes in segmental/subsegmental bronchi [7], which is fully consistent with the characteristic pathological changes of MOB.

In addition, this study found no direct correlation between HLA matching and the severity of MOB. Although Patient 2 received an HLA-matched transplant, MOB progressed more rapidly due to multi-organ GVHD and severe immune imbalance.

#### Infection as an important driver of disease progression

Infection plays a dual role in post-HSCT MOB: a consequence of immunosuppression and an accelerator of disease progression. Patient 1 had a mixed infection of Aspergillus fumigatus and cytomegalovirus. Patient 2 only had a single infection with Pneumocystis jirovecii, resulting in relatively slow MOB progression. This indicates that early screening and control of opportunistic infections are key to delaying MOB progression.

### Clinical features and diagnostic key points of MOB after HSCT

#### Non-specific clinical manifestations prone to missed and misdiagnosis

The core symptoms of MOB include chronic cough, dyspnea on exertion, and wheezing. Severe large airway obstruction may present with inspiratory three-depression sign, and dry rales can be heard on lung auscultation, with moist rales in cases of infection. These symptoms are highly similar to those of BO and asthma. Both pediatric patients in this study presented the above typical manifestations after HSCT and during the course of GVHD, suggesting that for patients with post-HSCT GVHD (especially multi-organ GVHD), once unexplained dyspnea develops after HSCT, MOB should be included in the differential diagnosis to avoid misdiagnosis as simple infection or BO. Both patients in this study had childhood onset, suggesting that MOB after HSCT may be related to multiple factors such as children’s airway anatomical development, immune development, epithelial injury repair, and susceptibility to infection.

#### Non-characteristic changes on imaging and pulmonary function tests

Previous reports have stated that the typical imaging finding of MOB is atelectasis [8], but no atelectasis was observed in either case of this study, which may be attributed to lesions located in the distal segmental/subsegmental bronchi with thin membranous tissue. Bronchiectasis was a common CT feature in both cases, presumably caused by increased pressure in proximal bronchi and secondary dilatation due to distal airway occlusion, and concurrent pulmonary infection may also exacerbate bronchiectasis. In terms of pulmonary function, bronchiolitis obliterans typically presents with obstructive or mixed ventilatory dysfunction [8]. Both cases showed severe obstructive or mixed ventilatory dysfunction with negative bronchodilator test, showing no difference from the pulmonary function manifestations of BO, thus cannot be used as a differential basis. In addition, neither patient received regular pulmonary function monitoring, and pulmonary function was severely impaired at symptom onset, indicating that pulmonary function reexamination every 3 months within 2 years after HSCT is an important method for early detection of airway lesions.

#### Fiberoptic bronchoscopy as the gold standard for MOB diagnosis

Pathological diagnosis of MOB relies on large-specimen lung biopsy, which is clinically difficult to perform; sampling by TBLC and percutaneous lung biopsy is limited. Biopsies in both cases of this study only indicated chronic inflammation and could not confirm the diagnosis. Direct observation under fiberoptic bronchoscopy is currently the only reliable method for clinical diagnosis of MOB, characterized by membranous stenosis or occlusion of segmental and subsegmental bronchi (3rd–4th order), which can also exclude other obstructive etiologies such as foreign body, external compression, tuberculosis, and tumor. Both cases in this study were diagnosed with MOB based on typical bronchoscopic findings, highlighting the irreplaceable role of fiberoptic bronchoscopy in MOB diagnosis.

### Differential diagnosis

Due to the lack of specificity in symptoms, imaging, and pulmonary function, MOB needs to be differentiated from various obstructive airway diseases. The core differential points are summarized as follows:

- Bronchiolitis Obliterans (BO/BOS): Involves small airways < 2 mm, pathologically characterized by fibrotic occlusion of bronchioles. BOS is a clinical diagnosis based on pulmonary function and CT air trapping, without large airway membranous occlusion under bronchoscopy. The two may coexist, and differentiation requires bronchoscopy combined with pathology. Pathological examination revealed no bronchiolar occlusion in both patients in this study, thus the diagnosis was excluded.
- Endobronchial Tuberculosis: Accompanied by tuberculous toxic symptoms such as low-grade fever and night sweats. Granulomas and cicatricial stenosis can be seen under bronchoscopy, with caseous necrosis on pathology and positive tuberculosis etiology. No relevant manifestations were found in either case of this study, thus excluded.
- Airway-Centered Interstitial Pulmonary Fibrosis: Mostly associated with smoking or occupational exposure, presenting mainly restrictive pulmonary dysfunction. CT shows airway-centered reticular fibrosis without luminal membranous occlusion, inconsistent with the cases in this study.
- Plastic Bronchitis: Mostly secondary to infection, with dendritic cast foreign bodies visible under bronchoscopy, and symptoms relieved after removal. No characteristic casts were found in this study, thus excluded.
- Iatrogenic Airway Cicatricial Stenosis: History of tracheal intubation or tracheotomy, with stenosis mostly located in the main bronchi rather than segmental/subsegmental bronchi. No relevant history was found in this study, thus excluded.
- Bronchial Asthma and Bronchial Foreign Body: Asthma is associated with allergy history and positive bronchodilator test; foreign body has a clear aspiration history and can be directly detected under bronchoscopy. Neither was supported in this study.
- Granulomatosis with Polyangiitis (GPA): A systemic autoimmune vasculitis that frequently involves the upper and lower respiratory tracts. Patients often present with sinusitis, hemoptysis, renal impairment, and positive ANCA serology. Bronchoscopy may reveal airway granulomas and ulcerative lesions, while membranous luminal occlusion limited to segmental/subsegmental bronchi is uncommon. No sinus, renal or ANCA abnormalities were observed in our two patients, so this diagnosis was excluded.
- Relapsing Polychondritis: An autoimmune disease targeting cartilaginous tissues, predominantly affecting tracheobronchial cartilages, auricles and nasal cartilage. Typical manifestations include tracheobronchial cartilage thickening, softening or collapse on CT/bronchoscopy, accompanied by recurrent chondral inflammation elsewhere. No cartilage injury or extra-pulmonary chondritis manifestations were identified in this cohort, thus ruled out.

### Treatment strategies and prognostic implications

Post-HSCT MOB has an extremely poor prognosis. Both patients in this study died despite active treatment. Clinical treatment requires a comprehensive regimen that may include controlling immune injury, maintaining airway patency, and implementing supportive care.

#### Immunosuppressive therapy

The pathogenesis of MOB remains unclear, and there is no standardized therapeutic regimen for this disease; The above findings indicate that the pathogenesis of MOB is associated with GVHD progression, immune dysregulation and infectious triggers. Early initiation of intensive immunosuppressive therapy is recommended for suspected MOB cases. The following regimens represent potential treatment options for clinical reference.

Intravenous methylprednisolone should be administered based on the patient’s infectious status, combined with immunosuppressants including tacrolimus and mycophenolate mofetil. Targeted agents such as rituximab can be added for patients refractory to glucocorticoid treatment.

Throughout the entire treatment period, infection risks require close monitoring. Prophylactic antifungal and antiviral medications should be given simultaneously to reduce the incidence of opportunistic infections.

#### Bronchoscopic interventional therapy

For membranous stenosis/occlusion detected under bronchoscopy, early interventional therapy (balloon dilation, biopsy forceps recanalization, laser ablation) can delay complete airway occlusion and improve ventilation. However, interventional procedures require strict risk assessment to avoid aggravating airway injury.

#### Supportive care and salvage treatment

Aerosol inhalation therapies including inhaled corticosteroids and bronchodilators may exert therapeutic effects. Oxygen inhalation and non-invasive/invasive ventilation are administered to improve respiratory failure, with ECMO support if necessary. For patients with end-stage lung disease, lung transplantation is the only radical treatment, but strict evaluation of transplantation indications is required. Despite comprehensive treatment, the prognosis of the cases in this study remained poor, indicating that once MOB progresses to severe airway occlusion, the therapeutic effect is extremely poor, and early identification and intervention are the core to improving prognosis.

### Research summary

Post-HSCT MOB lacks specific clinical manifestations, and large airway membranous occlusion under bronchoscopy is the key to diagnosis. Treatment may consist of immunosuppression combined with interventional therapy, with an extremely poor prognosis. This case series adds to the literature for pulmonary complications that may be related to HSCT. Clinicians should enhance awareness of MOB, and perform bronchoscopy as early as possible for dyspneic patients with post-HSCT GVHD to achieve early diagnosis and treatment.

## Conclusion

Post-HSCT MOB is clinically rare and prone to missed and misdiagnosis. Its pathogenesis may be GVHD progression, immune dysregulation and infectious triggers. Fiberoptic bronchoscopy is the preferred diagnostic method for MOB. Comprehensive interventions targeting GVHD, infection, and airway occlusion are required clinically, and early screening and timely diagnosis are crucial to improving patient prognosis.

## Data Availability

The raw data related to this study can be reasonably requested via email from the corresponding author of the article, and all core data used for conclusion analysis are fully presented in the published content of this paper.

## Abbreviations

HSCT: Hematopoietic stem cell transplantation
MOB: Membranous obliterans bronchitis
BO: Bronchiolitis obliterans
BOS: Bronchiolitis obliterans syndrome
GVHD: Graft-versus-host disease
cGVHD: Chronic graft-versus-host disease
aGVHD: Acute graft-versus-host disease
CT: Computed tomography
PFT: Pulmonary function test
FEV1: Forced expiratory volume in one second
FVC: Forced vital capacity
BALF: Bronchoalveolar lavage fluid
mNGS: Metagenomic next-generation sequencing
TBLC: Transbronchial lung cryobiopsy
CMV: Cytomegalovirus
